# Heterogeneous and Dynamic Prevalence of Asymptomatic Influenza Virus Infections

**DOI:** 10.3201/eid2206.151080

**Published:** 2016-06

**Authors:** Luis Furuya-Kanamori, Mitchell Cox, Gabriel J. Milinovich, Ricardo J. Soares Magalhaes, Ian M. Mackay, Laith Yakob

**Affiliations:** Australian National University, Acton, Australian Capital Territory, Australia (L. Furuya-Kanamori);; University of Queensland, Herston, Queensland, Australia (M. Cox, G.J. Milinovich, R.J. Soares Magalhaes);; Queensland University of Technology, Kelvin Grove, Queensland, Australia (I.M. Mackay);; London School of Hygiene and Tropical Medicine, London, UK (L. Yakob)

**Keywords:** flu, influenza, subclinical, pandemic, symptomless proportion, asymptomatic, meta-analysis, prevalence

## Abstract

Influenza infection manifests in a wide spectrum of severity, including symptomless pathogen carriers. We conducted a systematic review and meta-analysis of 55 studies to elucidate the proportional representation of these asymptomatic infected persons. We observed extensive heterogeneity among these studies. The prevalence of asymptomatic carriage (total absence of symptoms) ranged from 5.2% to 35.5% and subclinical cases (illness that did not meet the criteria for acute respiratory or influenza-like illness) from 25.4% to 61.8%. Statistical analysis showed that the heterogeneity could not be explained by the type of influenza, the laboratory tests used to detect the virus, the year of the study, or the location of the study. Projections of infection spread and strategies for disease control require that we identify the proportional representation of these insidious spreaders early on in the emergence of new influenza subtypes or strains and track how this rate evolves over time and space.

## Abstract 2

Extreme heterogeneity was found within and between influenza types, which should be considered in planning of mitigation campaigns.

Infection of the respiratory tract with an influenza virus results in symptoms ranging from mild nonfebrile illness to severe disease and complications, including pneumonia, shock, renal failure, encephalopathy, and multiorgan dysfunction (*1*,*2*). Influenza viruses infect 5%–15% of the global population annually (*3*), accounting for ≈500,000 deaths (*4*) and 19 million disability-adjusted life years (*5*). The occurrence of asymptomatic influenza viruses infections has been recognized for some time (*6*), but determinations about their possible role in transmission are largely speculative (*7*,*8*). Clarifying the role of these infections in virus transmission requires a solid understanding of their rate of occurrence.

Interest in the contribution of asymptomatic infection to influenza virus transmission has risen in recent years after a series of outbreaks caused by newly emerging subtypes (*9*–*12*). Subclinical infection eludes symptomatic surveillance, and resulting illnesses thus manifest as sporadic disease. Social network analysis indicates that nearly one third of the attack rate for influenza A(H1N1)pdm09 virus in England was attributable to asymptomatic infection (*13*), a proportion mirrored by a recent review of volunteer challenge studies (*14*). Mathematical modeling studies designed to inform pandemic preparedness and vaccination thresholds and stockpiling strategies have typically had to resort to using these types of indirect metrics for parameterization (*15*–*17*). Current policy surrounding intervention planning for pandemic and interpandemic influenza is informed by estimates and simulations that arbitrarily assume a constant rate of asymptomatic infection in the range of 30%–50%.

However, mortality rates, clinical symptoms, and basic reproduction numbers (outbreak thresholds) vary greatly between influenza virus types, subtypes, and strains (*18*). Therefore, assigning an arbitrary value for asymptomatic infection rates that does not reflect this heterogeneity presents an important shortcoming in the current ability to accurately predict influenza outbreaks. Therefore, we conducted a systematic review and meta-analysis to determine the prevalence of asymptomatic influenza infection and to identify any factors associated with the heterogeneity reported across studies.

## Methods

### Search Strategy and Selection Criteria

A systematic review and meta-analysis was conducted in accordance with PRISMA (Preferred Reporting Items for Systematic Reviews and Meta-Analyses) guidelines (*19*). Literature searches were performed on the PubMed and Web of Science databases for the period from the inception of these databases to the beginning of 2015 to identify studies that reported laboratory-confirmed influenza infection (i.e., by culture, PCR, or serologic testing) and the proportion of symptomatic versus asymptomatic presentation. Search terms were chosen to ensure maximum coverage of possible literature and included the terms “influenza,” “carrier,” “carriage,” “shedding,” “asymptomatic,” “influenza AND prophylaxis NOT vaccine” (filtered for randomized control trials), “influenza AND (travel OR migration OR immigra*) AND (screening OR test OR testing OR detection),” “subclinical,” “serosurvey OR seroprevalence OR seroepidemiology.” Other keywords and connectors were also used (Technical Appendix 1).

To be eligible for inclusion, studies needed to 1) be peer-reviewed and 2) report the prevalence of asymptomatic influenza virus infections in humans or present the appropriate data from which that prevalence could be calculated. Laboratory confirmation of influenza was a requirement, and it had to be possible to correlate these data to the number of symptomatic patients. We did not impose limitations in terms of study design, influenza virus type, or exposure type (community or experimental inoculation). According to current World Health Organization guidelines, laboratory confirmation consisted of 1) conventional PCR (referred to here as PCR) or real-time reverse transcription PCR (rRT-PCR); 2) virus antigen detection by immunofluorescence or enzyme immunoassay methods; 3) serologic detection of antibodies (hemagglutination inhibition); or 4) virus culture (*20*). Studies were excluded when the use of antiviral agents without a placebo group was reported. In cases in which a placebo group was used and an asymptomatic proportion could be determined, only this subset was used; otherwise, the study was excluded. Results were restricted to studies published in English; however, no restriction was placed on the publication date of studies that fit these criteria.

### Study Selection and Data Extraction

Two authors (L.F.-K. and M.C.) independently screened the publications for eligibility in a stepwise fashion. Search results were initially screened based on article titles and abstracts. Then, full-text analysis was performed to identify all studies which either reported asymptomatic prevalence or from which asymptomatic prevalence could be calculated. Any discrepancies that might have affected inclusion or exclusion of a study were resolved through discussion and consensus after independent evaluation by another author (L.Y.). The same 2 authors (L.F.-K. and M.C.) assessed the risk for bias of the studies included by using a modified version of the tool developed by Hoy et al. (*21*) for prevalence studies (Technical Appendix 2).

The definitions of asymptomatic influenza infection varied considerably between studies. Definitions ranged from a total absence of symptoms to a lack of influenza-like illness (ILI) or acute respiratory illness (ARI). For the sake of clarity, we used the term “asymptomatic” when there was a total absence of symptoms and “subclinical” when the patient did not meet the authors’ criteria for ILI or ARI. Asymptomatic influenza prevalence was considered to be the proportion of all persons with laboratory-confirmed influenza who had no symptoms, whereas subclinical influenza prevalence was the proportion of persons with laboratory-confirmed influenza who failed to meet the study’s definition of symptomatic infection. In addition to collecting data on asymptomatic and subclinical infection prevalence, we collected data on influenza virus type/subtype and study characteristics (e.g., study design, sample size, diagnostic test used to detect influenza virus infection, and the working definition of “symptomatic”).

### Statistical Analysis

We used prevalence of asymptomatic versus subclinical carriers among persons with laboratory-confirmed influenza as primary endpoints of interest. We pooled the prevalence estimates of asymptomatic and subclinical influenza across studies by using 2 meta-analytical models, the inverse variance heterogeneity model (*22*) and the random effects model.

We observed considerable heterogeneity across studies. This heterogeneity was unlikely to be attributable only to random or systematic errors, and actual clinical heterogeneity was deemed to exist. Therefore, we created subgroups by influenza virus type/subtype with the aim of generating more homogeneous groups within which we could anticipate that the differences indeed reflected variability caused by random or systematic error rather than actual clinical heterogeneity. In addition, we built a linear model to examine the variance explained by the influenza virus type/subtype, laboratory test used to detect the virus, year of the study, and geographic location of the study to gain insight into the considerable heterogeneity observed in the prevalence of asymptomatic and subclinical infections. We conducted the meta-analyses by using MetaXL version 2.0 (EpiGear Int Pty Ltd, Brisbane, QLD, Australia), which also included the inverse variance heterogeneity method, and the generalized linear model by using Stata version 12 (StataCorp LP, College Station, TX, USA). All tests were 2-tailed, and a p value <0.05 was deemed statistically significant. 

## Results

### Yield of Search Strategy

A total of 13,219 records were identified from literature searches of the 2 databases. This number was reduced to 9,900 after removal of publications that were either duplicates or not original research papers (e.g., review papers). An additional 3,663 papers were removed based on the title and 5,652 papers more based on the abstract. The full texts of the remaining 585 studies were examined, and 55 articles met the inclusion criteria and were included in the final analysis (Figure; Technical Appendix 1).

**Figure:**
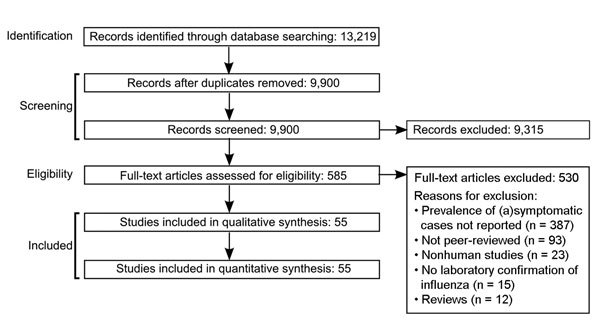
PRISMA (Preferred Reporting Items for Systematic Reviews and Meta-Analysis) flowchart of literature search for systematic review and meta-analysis of asymptomatic and subclinical influenza infection prevalence.

### Characteristics of the Studies Included

The 55 articles provided 59 data points because 4 papers reported the prevalence of asymptomatic and subclinical carriers for influenza A and B viruses separately. Overall, 19 studies (22 data points) defined asymptomatic infection as cases in persons lacking symptoms, and 44 studies (46 data points) reported subclinical influenza virus infections.

Infection was confirmed by serologic testing, rRT-PCR, or viral culture; 28 studies reported use of serologic testing alone to confirm infection, 18 used rRT-PCR alone, and the remaining 9 used a combination of methods (5 serologic testing and rRT-PCR, 3 serologic testing and culture, and 1 rRT-PCR and culture). Among the 55 studies, influenza A virus (predominantly H1N1) was the most common type of infection; 5 studies reported influenza B virus infections, and 1 study reported influenza C infections (Technical Appendix 1 Table 2). Most studies reported on pandemic influenza virus types; 32 of these studies related to the 2009 pandemic influenza A/Mexico/4108/2009 strain. The risk for bias was moderate in 32% of the studies and low in the remaining 68%; no study was found to have a high risk for bias.

### Quantitative Synthesis

The overall pooled prevalence for asymptomatic carriers was 19.1% (95% CI 5.2%–35.5%) for any type of influenza, 21.0% (95% CI 4.2%–41.0%) for influenza A, and 22.7% (95% CI 7.7%–39.8%) for influenza A(H1N1) (Table 1; Technical Appendix 2 Figure 1). For subclinical carriers, the overall pooled prevalence was 43.4% (95% CI 25.4%–61.8%) for any type of influenza, 42.8% (95% CI 22.3%–63.9%) for influenza A, and 39.8% (95% CI 16.4%–64.5%) for influenza A(H1N1) (Table 1; Technical Appendix 2 Figure 2). However, extensive heterogeneity was immediately evident for reported asymptomatic prevalence (τ^2^ = 0.31) and subclinical prevalence (τ^2^ = 0.45) that could not be explained by the influenza type/subtype alone. Similar results were obtained with the random effects model (Technical Appendix 2 Figures 3, 4).

**Table 1:** Heterogeneity within asymptomatic and subclinical influenza infection cases, by virus type/subtype, as determined through a systematic review and meta-analysis of 55 studies

Type/subtype	Prevalence (95% CI)	Cochran’s Q	p value (Cochran’s Q)	I^2^,* %
Asymptomatic				
All types of influenza	19.1 (5.2–35.5)	752.40	<0.001	97
Influenza A	21.0 (4.2–41.0)	692.94	<0.001	98
Influenza A(H1N1)	22.7 (7.7–39.8)	561.14	<0.001	97
Subclinical				
All types of influenza	43.4 (25.4–61.8)	1768.24	<0.001	97
Influenza A	42.8 (22.3–63.9)	1689.78	<0.001	98
Influenza A(H1N1)	39.8 (16.4–64.5)	1388.54	<0.001	98

*The I² statistic describes the percentage of variation across studies that is attributable to heterogeneity rather than chance.

### Investigation of Heterogeneity

The considerable heterogeneity observed within asymptomatic and subclinical influenza prevalence could not be explained by the type/subtype of influenza, the laboratory tests used to detect the virus, the location where the study was conducted, or the year of the study. The multivariate regression models could only explain 16.8% and 14.8% of the observed variance for the asymptomatic and subclinical prevalence, respectively. Influenza type/subtype as an independent predictor was found to account for almost the entire variance (16%) found for the prevalence of asymptomatic carriers (Table 2).

**Table 2:** Variance attributable to predictors in univariate and multivariate regression models for asymptomatic and subclinical influenza infection prevalence, by study characteristics, as determined through a systematic review and meta-analysis of 55 studies

Model/characteristic	Asymptomatic	Subclinical
Univariate model		
Influenza type/subtype	0.1599	0.0345
Laboratory test used to detect influenza	0.0043	0.0546
Hemisphere where study was conducted	0.0001	0.0159
Continent where study was conducted	0.0045	0.0213
Decade when study was conducted	*	0.0064
Multivariate model	0.1676†	0.1478‡

*Variance not reported because all the studies were from the same decade. †Model adjusted for influenza type/subtype, laboratory test, and location (continent) of the study. ‡Model adjusted for influenza type/subtype, laboratory test, location (continent) of the study, and decade when the study was conducted.

### Publication Bias

For both asymptomatic and subclinical carrier prevalence, the funnel plots showed no indication of publication bias. This result was confirmed by Doi plots (data not shown). 

## Discussion

Studies of laboratory-confirmed influenza typically do not include details of the symptomatic versus asymptomatic rate of infection. Of the few that do include this information, ambiguity exists between definitions of asymptomatic versus subclinical infections. This has perpetuated the ubiquitous issue of absent denominators in documented influenza rates and has caused substantial aberrations in initial reports of newly emerging subtypes and strains (*23*). We propose that the term “asymptomatic” be used exclusively to describe the complete absence of symptoms associated with influenza virus infection in patients with laboratory-confirmed cases. Given that reporting of this rate in the clinical literature would require little to no additional effort for most study designs, we also propose that the asymptomatic rate of laboratory test–positive persons be declared explicitly by public health bodies and researchers.

We found no evidence to support a fixed asymptomatic rate (or even an informative range) between or even within influenza virus subtypes. For example, the prevalence of asymptomatic influenza A(H1N1) virus ranged from 0% to 65%, resulting in an overall failure to explain the extreme heterogeneity in this reported rate. Some alternative explanations for the extreme heterogeneity are plausible, one being that generally applicable biologic mechanisms underlie the asymptomatic rates of influenza virus infection and these have been missed (e.g., details of patient vaccination or infection history were not routinely described in the clinical studies and data on sex and age of patients were excluded). Alternatively, influenza viruses conferring asymptomatic infection mutate so rapidly that a meaningful single per–influenza type rate simply does not exist. Employing sensitive diagnostic testing and standardized reporting of the asymptomatic rate of influenza virus infection would elucidate any underlying mechanisms or demonstrate any temporal changes in this rate.

This lack of a convenient asymptomatic rate poses a considerable obstacle to public health planning. Disease surveillance and control strategy is contingent on reliable estimates for the asymptomatic rate and the contribution that asymptomatic persons have on transmissibility. For example, a low asymptomatic rate improves the utility of passive (i.e., symptom-based) surveillance, whereas a higher asymptomatic rate might prompt presumptive travel restrictions to curb the spread of newly emerging subtypes and strains, especially if a high mortality rate is evident early in the outbreak. Future analyses correlating asymptomatic rates with mortality rates are also required; although one could easily speculate that influenza subtypes and strains eliciting high asymptomatic rates probably incur correspondingly low mortality rates, no evidence supporting this assumption currently exists.

Our study clearly demonstrates the inappropriateness of a one-size-fits-all approach to mitigating the spread of human influenza viruses. As new subtypes and strains emerge, actively surveying infection status of local populations and tracking any changes in asymptomatic rates of infection should increasingly become a global health priority, possibly necessitating the provision of international resources and the deployment of dedicated rapid-response teams who are guided by standardized protocols.

Technical Appendix 1 Study selection and characteristics of the 55 studies included in systematic review and meta-analysis of asymptomatic and subclinical influenza infection prevalence.

Technical Appendix 2 Risk for bias assessment, statistical analysis, and forest plots for systematic review and meta-analysis of asymptomatic and subclinical influenza infection prevalence.
