# Supplementary material for: Heterogeneous and Dynamic Prevalence of Asymptomatic Influenza Virus Infections
Source: Emerg Infect Dis. 2016 Jun;22(6):1052–6. doi: 10.3201/eid2206.151080 (PMC4880086; doi:10.3201/eid2206.151080)
Supplement: Technical Appendix 1 — Study selection and characteristics of the 55 studies included in systematic review and meta-analysis of asymptomatic and subclinical influenza infection prevalence. [file 15-1080-Techapp-s1.pdf]

Article DOI: http://dx.doi.org/10.3201/eid2206.151080

Heterogeneous and Dynamic Prevalence of Asymptomatic Influenza Virus Infections

Technical Appendix 1

Search Strategies

First Search

Influenza AND ((Asymptomatic OR Carrier OR carriage OR shedding OR symptomatic OR Subclinical OR serosurvey OR seroprevalence OR seroepidemiology) OR ((travel OR migration OR immigra*) AND (screening OR test OR testing OR detection)) OR ((“Cohort Studies”[Mesh] OR “Case-Control Studies”[Mesh]) AND “Influenza A virus”[Mesh]))

Second Search

(“influenza, human”[MeSH Terms] OR (“influenza”[All Fields] AND “human”[All Fields]) OR “human influenza”[All Fields] OR “influenza”[All Fields]) AND (“prevention and control”[Subheading] OR (“prevention”[All Fields] AND “control”[All Fields]) OR “prevention and control”[All Fields] OR “prophylaxis”[All Fields]) NOT (“vaccines”[MeSH Terms] OR “vaccines”[All Fields] OR “vaccine”[All Fields])

Manually filtered for randomized controlled trials.

Technical Appendix Table 1. Characteristics of the 55 studies included in systematic review and meta-analysis of asymptomatic and subclinical influenza infection prevalence

Authors, year	Location of the study	Influenza type or subtype	Seasonal / Pandemic	Exposure type	Diagnosis test	Definition of asymptomatic	Definition of subclinical	Asymptomatic prevalence, %	Subclinical prevalence, %
Aho M, et al., 2010	Finland	A (H1N1)	Pandemic	Military garrison	HI ≥10	No symptoms of URT infection	—	40.7	—
Belderok SM, et al., 2013	Netherlands	A, and B	Seasonal	Travel to tropical and subtropical countries	HI ≥40 and ≥4-fold increase above pre-travel titer	—	No ILI	—	90.83
Bone A, et al., 2012	France	A (H1N1)	Pandemic	Community	HI ≥40	—	No ILI	—	29.52
Buescher, et al., 1969	Thailand and Panama	A (H3N1)	Pandemic	Military garrison	HI ≥32	—	No ILI	—	77.5
Carey DE, et al., 1958	USA	A (H2N2)	Pandemic	Parish	HI ≥10	—	No ‘flu’	—	24.77
Ceyhan M, et al., 2010	Turkey	A (H5N1)		Community, poultry exposure and healthcare workers	HI ≥21	—	No symptoms of avian influenza infection	—	81.25
Clover RD, et al., 1986*	USA	A (H1N1)	Seasonal	Community	Positive culture or HI ≥4-fold increase	—	No ILI	—	60
Cui F, et al., 2011	China	A (H1N1)	Pandemic	Train	rRT-PCR	—	No ARI	—	13.64
Dotan A, et al., 2014	Israel	A (H1N1)	Pandemic	Hospital	rRT-PCR	—	No URI	—	30.77
Du Ry van Beest Holle M, et al., 2005	Netherlands	A (H7N7)		Poultry	HI ≥10	—	No ILI	—	93.94
Foy HM, et al., 1987	USA	B	Seasonal	Community	HI ≥10	—	No ILI	—	32.43
Gray GC, et al., 2014	Cambodia	A (H1N1), A (H3N2), and B	Seasonal and pandemic	Community	HI ≥4-fold increase	—	No ILI	—	64.44
Guinard A, et al., 2009	France	A (H1N1)	Pandemic	School	rRT-PCR	—	No ILI	—	53.33
Hayden FG, et al., 1999*	USA	A (H1N1)	Seasonal	Experimental inoculation	Positive culture and/or HI ≥4-fold increase	—	No URT illness	—	46.15
Hayward AC, et al., 2014	UK	A (H1N1), A (H3N2), and B	Seasonal and pandemic	Community	rRT-PCR	—	No ILI	—	46.22
Hsieh YH, et al., 2014	Taiwan	A (H1N1)	Seasonal	Community and school	HI ≥4-fold increase	No symptoms	No ILI	45.15	33.33
Hudson L, et al., 2013	New Zealand	A (H1N1)	Pandemic	Healthcare workers	HI ≥40	None influenza symptoms	—	25.44	—
Ison MG, et al., 2012*	Belgium, Estonia, France, Germany, Hungary, Israel, Italy, Lithuania, Spain, UK, USA	A (H1N1), A (H3N2), and B		Transplant recipients	Positive culture and/or HI ≥4-fold increase	No symptoms	—	25	—
Jackson ML, et al., 2011	USA	A (H1N1)	Pandemic	School	HI ≥20 and ≥4-fold increase	No symptoms	No ILI	25	81.25

Authors, year	Location of the study	Influenza type or subtype	Seasonal / Pandemic	Exposure type	Diagnosis test	Definition of asymptomatic	Definition of subclinical	Asymptomatic prevalence, %	Subclinical prevalence, %
Jaeger JL, et al., 2011	USA	A (H1N1)	Pandemic	Hospital	HI ≥20	—	No ARI or ILI	—	66.66
Johnson S, et al., 2011	UK	A (H1N1)	Pandemic	Boarding school	HI ≥8	—	No ILI	—	68.35
Khakpour M, et al., 1969	Iran	A (H3N2)	Pandemic	Prisoners	HI	—	No ILI	—	23.53
Khaokham CB, et al., 2013	USA	A (H1N1)	Pandemic	Navy vessel	rRT-PCR or HI ≥4-fold increase	No symptoms	No ILI	52.11	88.03
Khuntirat B, et al., 2014	Thailand	A (H1N1)	Pandemic	Community	rRT-PCR and HI ≥4-fold increase	—	No ILI	—	83.33
Kumar S, et al., 2010	USA	A (H1N1)	Pandemic	Community	rRT-PCR	No symptoms	No ILI	10	32
Kumar S, et al., 2011	USA	A (H1N1)	Pandemic	Healthcare workers	HI ≥40	No symptoms	No ILI	35	30
Kuster SP, et al., 2013	Canada	A (H1N1)	Pandemic	Community and healthcare workers	HI ≥40	—	No ARI	—	13.04
Lau LLH, et al., 2010	Hong Kong	A (H1N1), A (H3N2), and B	Seasonal	Community	rRT-PCR	No symptoms	—	25.42	—
Levy JW, et al., 2013	Thailand	A (H1N1), A (H3N2), and B	Seasonal and pandemic	Community	rRT-PCR	No symptoms	—	2.54	—
Li T, et al., 2011	China	A (H1N1)	Pandemic	Boarding school	rRT-PCR and HI ≥40	No symptoms	—	30.89	—
Mikulska M, et al., 2013	Italy	A (H1N1), A (H3N2), and B	Seasonal	Allogeneic haematopoietic stem cell recipients	rRT-PCR	No symptoms	No ILI	10	45
Neatherlin J, et al., 2013	USA	A (H1N1)	Pandemic	Airplane	MN ≥40 and HI ≥20	—	No ARI/ILI	—	75
Oker-Blom N, et al., 1970*	Finland	A (H3N2)	Pandemic	Community	HI ≥4-fold increase	—	No respiratory illness	—	18
Pang X, et al., 2011	China	A (H1N1)	Pandemic	Community	rRT-PCR	—	No ILI	—	4.62
Papenburg J, et al., 2010	Canada	A (H1N1)	Pandemic	Community	Microneutralization ≥40 or ≥4-fold increase	No symptoms	—	9.43	—
Pascalis H, et al., 2012	Reunion Island	A (H1N1)	Pandemic	Community	rRT-PCR	No symptoms	No ILI	1.61	30.65
Pasco JA, et al., 2012	Australia	A (H1N1)	Pandemic	Community	HI ≥40	—	No ILI	—	75.97
Paton NI, et al., 2011*	Singapore	A (H1N1), A (H3N2), and B	Seasonal	Community	HI ≥4-fold increase	—	No clinical influenza (ILI)	—	51.72
Priest PC, et al., 2013	New Zealand	A, and B	Seasonal	Airport	rRT-PCR	No symptoms	—	6.67	—
Qi W, et al., 2014	China	A (H10N8)	Pandemic	Poultry exposure	HI ≥20	—	No influenza symptoms	—	100
Redlberger-Fritz M, et al., 2014	Austria	A (H1N1)	Pandemic	Attended hospital	rRT-PCR	—	No respiratory symptoms	—	60.72

Authors, year	Location of the study	Influenza type or subtype	Seasonal / Pandemic	Exposure type	Diagnosis test	Definition of asymptomatic	Definition of subclinical	Asymptomatic prevalence, %	Subclinical prevalence, %
Robinson JL, et al., 2007	Canada	A (H3N2)		Community	HI ≥32	—	No ILI	—	77.78
Salez N, et al., 2014	France, Reunion Island, UK	C		Community	HI, ELISA and rRT-PCR	—	No ILI	—	50
Shafir SC, et al., 2011	USA	A (H1N1)		University campus	HI ≥40	—	No ILI	—	54.43
Shankar AG, et al., 2014	UK	A (H1N1)	Pandemic	Airplane	rRT-PCR	—	No ILI	—	0
Smit PM, et al., 2012	Netherlands	A (H1N1)	Seasonal	Healthcare workers	rRT-PCR	—	No ILI	—	0
Sridhar S, et al., 2014	UK	A (H1N1)	Pandemic	Community	HI ≥32	—	No ILI	—	84.15
Suess T, et al., 2012	Germany	A (H1N1), A (H3N2), and B	Seasonal and pandemic	Community	rRT-PCR	No symptoms	No ILI	4.76	17.99
Thai PQ, et al., 2014	Vietnam	A (H1N1)	Pandemic	Community	rRT-PCR	No symptoms	—	45.45	—
Toyokawa T, et al., 2011	Japan	A (H1N1)	Pandemic	Healthcare workers	HI ≥40	—	No fever	—	92.86
Vilella A, et al., 2012	Dominican Republic	A (H1N1)	Pandemic	Community	rRT-PCR	No symptoms	—	5.13	—
Wang TE, et al., 2010	Taiwan	A (H1N1), A (H3N2)	Seasonal	School	HI ≥4-fold increase	No symptoms	—	62.5	—
Woods CW, et al., 2013	USA and UK	A (H1N1), A (H3N2)	Seasonal	Experimental inoculation	Positive culture or rRT-PCR	—	Jackson score <6	—	56.1
Yan L, et al., 2012	China	A (H1N1)	Pandemic	School	rRT-PCR or HI ≥40	—	No ARI	—	64.49
Zaman M, et al., 2011	Pakistan	A (H5N1)	Pandemic	Hospital	rRT-PCR	No symptoms	—	25	—

*Only control or placebo group included; ARI, acute respiratory illness; HI, hemagglutination inhibition; ILI, influenza-like illness; LRT, lower respiratory tract; rRT-PCR, real-time reverse transcription PCR; URT, upper respiratory tract.

Technical Appendix 2 Table. Included studies (N = 55), by influenza type/subtype

Type	Subtype	No. of studies	References
A	H10N8	1	*1*
	H7N7	1	*2*
	H5N1	2	*3,4*
	H3N2	11	*5–15*
	H2N2	1	*16*
	H1N1	38	*5,8,9,12–15,17–47*
B		5	*5,8,12,48,49*
C		1	*50*
Mixed		6	*49,51–55*

References

1. Qi W, Su S, Xiao C, Zhou P, Li H, Ke C, et al. Antibodies against H10N8 avian influenza virus among animal workers in Guangdong Province before November 30, 2013, when the first human H10N8 case was recognized. BMC Med. 2014;12:205. PubMed http://dx.doi.org/10.1186/s12916-014-0205-3

2. Du Ry van Beest Holle M, Meijer A, Koopmans M, de Jager CM. Human-to-human transmission of avian influenza A/H7N7, The Netherlands, 2003. Euro Surveill. 2005;10:264–8. PubMed

3. Ceyhan M, Yildirim I, Ferraris O, Bouscambert-Duchamp M, Frobert E, Uyar N, et al. Serosurveillance study on transmission of H5N1 virus during a 2006 avian influenza epidemic. Epidemiol Infect. 2010;138:1274–80. PubMed http://dx.doi.org/10.1017/S095026880999166X

4. Zaman M, Ashraf S, Dreyer NA, Toovey S. Human infection with avian influenza virus, Pakistan, 2007. Emerg Infect Dis. 2011;17:1056–9. PubMed http://dx.doi.org/10.3201/eid/1706.091652

5. Belderok SM, Rimmelzwaan GF, van den Hoek A, Sonder GJ. Effect of travel on influenza epidemiology. Emerg Infect Dis. 2013;19:925–31. PubMed http://dx.doi.org/10.3201/eid1906.111864

6. Buescher EL, Smith TJ, Zachary IH. Experience with Hong Kong influenza in tropical areas. Bull World Health Organ. 1969;41:387–91. PubMed

7. Khakpour M, Saidi A, Naficy K. Proved viraemia in Asian influenza (Hong Kong variant) during incubation period. BMJ. 1969;4:208–9. PubMed http://dx.doi.org/10.1136/bmj.4.5677.208

8. Levy JW, Cowling BJ, Simmerman JM, Olsen SJ, Fang VJ, Suntarattiwong P, et al. The serial intervals of seasonal and pandemic influenza viruses in households in Bangkok, Thailand. Am J Epidemiol. 2013;177:1443–51. PubMed http://dx.doi.org/10.1093/aje/kws402

9. Neatherlin J, Cramer EH, Dubray C, Marienau KJ, Russell M, Sun H, et al. Influenza A(H1N1)pdm09 during air travel. Travel Med Infect Dis. 2013;11:110–8. PubMed http://dx.doi.org/10.1016/j.tmaid.2013.02.004

10. Oker-Blom N, Hovi T, Leinikki P, Palosuo T, Pettersson R, Suni J. Protection of man from natural infection with influenza A2 Hong Kong virus by amantadine: a controlled field trial. BMJ. 1970;3:676–8. PubMed http://dx.doi.org/10.1136/bmj.3.5724.676

11. Robinson JL, Lee BE, Patel J, Bastien N, Grimsrud K, Seal RF, et al. Swine influenza (H3N2) infection in a child and possible community transmission, Canada. Emerg Infect Dis. 2007;13:1865–70. PubMed http://dx.doi.org/10.3201/eid1312.070615

12. Suess T, Remschmidt C, Schink SB, Schweiger B, Heider A, Milde J, et al. Comparison of shedding characteristics of seasonal influenza virus (sub)types and influenza A(H1N1)pdm09; Germany, 2007-2011. PLoS One. 2012;7:e51653. PubMed http://dx.doi.org/10.1371/journal.pone.0051653

13. Wang TE, Lin CY, King CC, Lee WC. Estimating pathogen-specific asymptomatic ratios. Epidemiology. 2010;21:726–8. PubMed http://dx.doi.org/10.1097/EDE.0b013e3181e94274

14. Woods CW, McClain MT, Chen M, Zaas AK, Nicholson BP, Varkey J, et al. A host transcriptional signature for presymptomatic detection of infection in humans exposed to influenza H1N1 or H3N2. PLoS One. 2013;8:e52198. PubMed http://dx.doi.org/10.1371/journal.pone.0052198

15. Gray GC, Krueger WS, Chum C, Putnam SD, Wierzba TF, Heil GL, et al. Little evidence of subclinical avian influenza virus infections among rural villagers in Cambodia. PLoS One. 2014;9:e97097. PubMed http://dx.doi.org/10.1371/journal.pone.0097097

16. Carey DE, Dunn FL, Robinson RQ, Jensen KE, Martin JD. Community-wide epidemic of Asian strain influenza; clinical and subclinical illnesses among school children. J Am Med Assoc. 1958;167:1459–63. PubMed http://dx.doi.org/10.1001/jama.1958.02990290013004

17. Aho M, Lyytikaïnen O, Nyholm JE, Kuitunen T, Rönkkö E, Santanen R, et al. Outbreak of 2009 pandemic influenza A(H1N1) in a Finnish garrison—a serological survey. Euro Surveill. 2010;15:19709. PubMed

18. Bone A, Guthmann JP, Assal A, Rousset D, Degeorges A, Morel P, et al. Incidence of H1N1 2009 virus infection through the analysis of paired plasma specimens among blood donors, France. PLoS One. 2012;7:e33056. PubMed http://dx.doi.org/10.1371/journal.pone.0033056

19. Clover RD, Crawford SA, Abell TD, Ramsey CN Jr, Glezen WP, Couch RB. Effectiveness of rimantadine prophylaxis of children within families. Am J Dis Child. 1986;140:706–9. PubMed

20. Cui F, Luo H, Zhou L, Yin D, Zheng C, Wang D, et al. Transmission of pandemic influenza A (H1N1) virus in a train in China. J Epidemiol. 2011;21:271–7. PubMed http://dx.doi.org/10.2188/jea.JE20100119

21. Guinard A, Grout L, Durand C, Schwoebel V. Outbreak of influenza A(H1N1)v without travel history in a school in the Toulouse district, France, June 2009. Euro Surveill. 2009;14:19265. PubMed

22. Hayden FG, Treanor JJ, Fritz RS, Lobo M, Betts RF, Miller M, et al. Use of the oral neuraminidase inhibitor oseltamivir in experimental human influenza: randomized controlled trials for prevention and treatment. JAMA. 1999;282:1240–6. PubMed http://dx.doi.org/10.1001/jama.282.13.1240

23. Hudson B, Toop L, Mangin D, Brunton C, Jennings L, Fletcher L. Pandemic influenza A(H1N1)pdm09: risk of infection in primary healthcare workers. Br J Gen Pract. 2013;63:e416–22. PubMed http://dx.doi.org/10.3399/bjgp13X668212

24. Jackson ML, France AM, Hancock K, Lu X, Veguilla V, Sun H, et al. Serologically confirmed household transmission of 2009 pandemic influenza A (H1N1) virus during the first pandemic wave—New York City, April-May 2009. Clin Infect Dis. 2011;53:455–62. PubMed http://dx.doi.org/10.1093/cid/cir437

25. Jaeger JL, Patel M, Dharan N, Hancock K, Meites E, Mattson C, et al. Transmission of 2009 pandemic influenza A (H1N1) virus among healthcare personnel-Southern California, 2009. Infect Control Hosp Epidemiol. 2011;32:1149–57. PubMed http://dx.doi.org/10.1086/662709

26. Johnson S, Ihekweazu C, Hardelid P, Raphaely N, Hoschler K, Bermingham A, et al. Seroepidemiologic study of pandemic (H1N1) 2009 during outbreak in boarding school, England. Emerg Infect Dis. 2011;17:1670–7. PubMed http://dx.doi.org/10.3201/eid1709.100761

27. Khaokham CB, Selent M, Loustalot FV, Zarecki SM, Harrington D, Hoke E, et al. Seroepidemiologic investigation of an outbreak of pandemic influenza A H1N1 2009 aboard a US Navy vessel—San Diego, 2009. Influenza Other Respi Viruses. 2013;7:791–8. PubMed http://dx.doi.org/10.1111/irv.12100

28. Kumar S, Chusid MJ, Willoughby RE, Havens PL, Kehl SC, Ledeboer NA, et al. Epidemiologic Observations from Passive and Targeted Surveillance during the First Wave of the 2009 H1N1 Influenza Pandemic in Milwaukee, WI. Viruses. 2010;2:782–95. PubMed http://dx.doi.org/10.3390/v2040782

29. Kumar S, Fan J, Melzer-Lange M, Trost J, Havens PL, Willoughby RE, et al. H1N1 hemagglutinin-inhibition seroprevalence in Emergency Department Health Care workers after the first wave of the 2009 influenza pandemic. Pediatr Emerg Care. 2011;27:804–7. PubMed http://dx.doi.org/10.1097/PEC.0b013e31822c125e

30. Kuster SP, Coleman BL, Raboud J, McNeil S, De Serres G, Gubbay J, et al.; Working Adult Influenza Cohort Study Group. Risk factors for influenza among health care workers during 2009 pandemic, Toronto, Ontario, Canada. Emerg Infect Dis. 2013;19:606–15. PubMed http://dx.doi.org/10.3201/eid1904.111812

31. Li T, Liu Y, Di B, Wang M, Shen J, Zhang Y, et al. Epidemiological investigation of an outbreak of pandemic influenza A (H1N1) 2009 in a boarding school: serological analysis of 1570 cases. J Clin Virol. 2011;50:235–9. PubMed http://dx.doi.org/10.1016/j.jcv.2010.11.012

32. Pang X, Yang P, Li S, Zhang L, Tian L, Li Y, et al. Pandemic (H1N1) 2009 among quarantined close contacts, Beijing, People's Republic of China. Emerg Infect Dis. 2011;17:1824–30. PubMed http://dx.doi.org/10.3201/eid1710.101344

33. Papenburg J, Baz M, Hamelin MÈ, Rhéaume C, Carbonneau J, Ouakki M, et al. Household transmission of the 2009 pandemic A/H1N1 influenza virus: elevated laboratory-confirmed secondary attack rates and evidence of asymptomatic infections. Clin Infect Dis. 2010;51:1033–41. PubMed http://dx.doi.org/10.1086/656582

34. Pascalis H, Temmam S, Turpin M, Rollot O, Flahault A, Carrat F, et al. Intense co-circulation of non-influenza respiratory viruses during the first wave of pandemic influenza pH1N1/2009: a cohort study in Reunion Island. PLoS One. 2012;7:e44755. PubMed http://dx.doi.org/10.1371/journal.pone.0044755

35. Pasco JA, Nicholson GC, Brennan SL, Bennett KE, Dobbins AG, Athan E. The epidemiology of the first wave of H1N1 influenza pandemic in Australia: a population-based study. Open Public Health J. 2012;5:80–5. http://dx.doi.org/10.2174/1874944501205010080

36. Shafir SC, O'Keefe KA, Shoaf KI. Evaluation of the seroprevalence of influenza A(H1N1) 2009 on a university campus: a cross-sectional study. BMC Public Health. 2011;11:922. PubMed http://dx.doi.org/10.1186/1471-2458-11-922

37. Smit PM, Mulder JW, Ahdi M, Gerritsen R, Darma S, Smits PH, et al. Low attack rate of novel influenza A (H1N1) virus infection among healthcare workers: a prospective study in a setting

with an elaborated containment plan. Int Arch Occup Environ Health. 2012;85:163–70. PubMed http://dx.doi.org/10.1007/s00420-011-0652-5

38. Toyokawa T, Sunagawa T, Yahata Y, Ohyama T, Kodama T, Satoh H, et al. Seroprevalence of antibodies to pandemic (H1N1) 2009 influenza virus among health care workers in two general hospitals after first outbreak in Kobe, Japan. J Infect. 2011;63:281–7. PubMed http://dx.doi.org/10.1016/j.jinf.2011.05.001

39. Vilella A, Serrano B, Marcos MA, Serradesanferm A, Mensa J, Hayes E, et al. Pandemic influenza A(H1N1) outbreak among a group of medical students who traveled to the Dominican Republic. J Travel Med. 2012;19:9–14. PubMed http://dx.doi.org/10.1111/j.1708-8305.2011.00580.x

40. Yan L, Gao Y, Zhang Y, Tildesley M, Liu L, Zhang Y, et al. Epidemiological and virological characteristics of pandemic influenza A (H1N1) school outbreaks in China in 2009. PLoS One. 2012;7:e45898. PubMed http://dx.doi.org/10.1371/journal.pone.0045898

41. Dotan A, Ben-Shimol S, Fruchtman Y, Avni-Shemer Y, Kapelushnik J, Ben-Harush M, et al. Influenza A/H1N1 in pediatric oncology patients. J Pediatr Hematol Oncol. 2014;36:e271–4. PubMed http://dx.doi.org/10.1097/MPH.0000000000000043

42. Hsieh YH, Tsai CA, Lin CY, Chen JH, King CC, Chao DY, et al.; CIDER Research Team. Asymptomatic ratio for seasonal H1N1 influenza infection among schoolchildren in Taiwan. BMC Infect Dis. 2014;14:80. PubMed http://dx.doi.org/10.1186/1471-2334-14-80

43. Khuntirat B, Yoon IK, Chittaganpitch M, Krueger WS, Supawat K, Blair PJ, et al. High rate of A(H1N1)pdm09 infections among rural Thai villagers, 2009-2010. PLoS One. 2014;9:e106751. PubMed http://dx.doi.org/10.1371/journal.pone.0106751

44. Thai PQ, Mai Q, Welkers MRA, Hang NK, Thanh T, Dung VT, et al. Pandemic H1N1 virus transmission and shedding dynamics in index case households of a prospective Vietnamese cohort. J Infect. 2014;68:581–90. PubMed http://dx.doi.org/10.1016/j.jinf.2014.01.008

45. Redlberger-Fritz M, Hirk S, Buchinger D, Haberl R, Hell M, Perkmann-Nagele N, et al. Distinct differences in clinical manifestation and viral laboratory parameters between children and adults with influenza A(H1N1)pdm09 infection—a retrospective comparative analysis. J Med Virol. 2014;86:1048–55. PubMed http://dx.doi.org/10.1002/jmv.23912

46. Shankar AG, Janmohamed K, Olowokure B, Smith GE, Hogan AH, De Souza V, et al. Contact tracing for influenza A(H1N1)pdm09 virus-infected passenger on international flight. Emerg Infect Dis. 2014;20:118–20. PubMed http://dx.doi.org/10.3201/eid2001.120101

47. Sridhar S, Begom S, Bermingham A, Hoschler K, Adamson W, Carman W, et al. Incidence of influenza A(H1N1)pdm09 infection, United Kingdom, 2009-2011. Emerg Infect Dis. 2013;19:1866–9. PubMed http://dx.doi.org/10.3201/eid1911.130295

48. Foy HM, Cooney MK, Allan ID, Albrecht JK. Influenza B in households: virus shedding without symptoms or antibody response. Am J Epidemiol. 1987;126:506–15. PubMed

49. Lau LL, Cowling BJ, Fang VJ, Chan KH, Lau EH, Lipsitch M, et al. Viral shedding and clinical illness in naturally acquired influenza virus infections. J Infect Dis. 2010;201:1509–16. PubMed http://dx.doi.org/10.1086/652241

50. Salez N, Mélade J, Pascalis H, Aherfi S, Dellagi K, Charrel RN, et al. Influenza C virus high seroprevalence rates observed in 3 different population groups. J Infect. 2014;69:182–9. PubMed http://dx.doi.org/10.1016/j.jinf.2014.03.016

51. Ison MG, Szakaly P, Shapira MY, Kriván G, Nist A, Dutkowski R. Efficacy and safety of oral oseltamivir for influenza prophylaxis in transplant recipients. Antivir Ther. 2012;17:955–64. PubMed http://dx.doi.org/10.3851/IMP2192

52. Mikulska M, Del Bono V, Gandolfo N, Dini S, Dominietto A, Di Grazia C, et al. Epidemiology of viral respiratory tract infections in an outpatient haematology facility. Ann Hematol. 2014;93:669–76. PubMed http://dx.doi.org/10.1007/s00277-013-1912-0

53. Paton NI, Lee L, Xu Y, Ooi EE, Cheung YB, Archuleta S, et al. Chloroquine for influenza prevention: a randomised, double-blind, placebo controlled trial. Lancet Infect Dis. 2011;11:677–83. PubMed http://dx.doi.org/10.1016/S1473-3099(11)70065-2

54. Priest PC, Jennings LC, Duncan AR, Brunton CR, Baker MG. Effectiveness of border screening for detecting influenza in arriving airline travelers. Am J Public Health. 2013;103:1412–8. PubMed http://dx.doi.org/10.2105/AJPH.2012.300761

55. Hayward AC, Fragaszy EB, Bermingham A, Wang L, Copas A, Edmunds WJ, et al.; Flu Watch Group. Comparative community burden and severity of seasonal and pandemic influenza: results of the Flu Watch cohort study. Lancet Respir Med. 2014;2:445–54. PubMed http://dx.doi.org/10.1016/S2213-2600(14)70034-7
