# Supplementary material for: Heterogeneous and Dynamic Prevalence of Asymptomatic Influenza Virus Infections
Source: Emerg Infect Dis. 2016 Jun;22(6):1052–6. doi: 10.3201/eid2206.151080 (PMC4880086; doi:10.3201/eid2206.151080)
Supplement: Technical Appendix 2 — Risk for bias assessment, statistical analysis, and forest plots for systematic review and meta-analysis of asymptomatic and subclinical influenza infection prevalence. [file 15-1080-Techapp-s2.pdf]

Article DOI: http://dx.doi.org/10.3201/eid2206.151080

Heterogeneous and Dynamic Prevalence of Asymptomatic Influenza Virus Infections

Technical Appendix 2

Risk for Bias Assessment

Risk for bias of the studies was assessed by using a modified version of the tool developed by Hoy et al. for prevalence studies. The modified tool assessed the external and internal validity of the studies by 9 criteria: 1) the targeted population was a close representation of the national population; 2) the sampling frame was a true or close representation of the general population; 3) random selection was used to select the study population; 4) the likelihood of nonresponse bias was minimal; 5) the case definition of influenza infection was based on laboratory tests; 6) the data collected were reliable; 7) the method used to collect the data was the same for all subjects; 8) the numerator and denominator of the prevalence were based on all the study participants; and 9) the data were largely recorded directly from the participants.

Statistical Analysis

All prevalence estimates were pooled after double arcsine square root transformation and back-transformed for reporting (*1*). We did not use the standard random effects (RE) model (*2*) to pool because the latter is known to underestimate the statistical error and exacerbate publication bias (*3–5*). Therefore, the prevalence rates of asymptomatic and subclinical influenza across studies within the subgroups were pooled using the inverse variance heterogeneity (IVhet) model (*6*). This method uses a quasi-likelihood based variance structure without distributional assumptions and thus has coverage probabilities for the CI well within the 95% nominal level and has been documented to have better performance (lower mean squared error) when compared to the RE method (*7*). The results from the RE model have nevertheless been reported (Technical Appendix 2 Figures 3 and 4) for comparison purposes. Publication bias was assessed through Egger's linear regression and visual inspection of funnel and Doi plots (*8*).

References

1. Barendregt JJ, Doi SA, Lee YY, Norman RE, Vos T. Meta-analysis of prevalence. J Epidemiol Community Health. 2013;67:974–8. PubMed http://dx.doi.org/10.1136/jech-2013-203104

2. DerSimonian R, Laird N. Meta-analysis in clinical trials. Control Clin Trials. 1986;7:177–88. PubMed http://dx.doi.org/10.1016/0197-2456(86)90046-2

3. Noma H. Confidence intervals for a random-effects meta-analysis based on Bartlett-type corrections. Stat Med. 2011;30:3304–12. PubMed http://dx.doi.org/10.1002/sim.4350

4. Senn S. Trying to be precise about vagueness. Stat Med. 2007;26:1417–30. PubMed http://dx.doi.org/10.1002/sim.2639

5. Poole C, Greenland S. Random-effects meta-analyses are not always conservative. Am J Epidemiol. 1999;150:469–75. PubMed http://dx.doi.org/10.1093/oxfordjournals.aje.a010035

6. Doi SA, Barendregt JJ, Khan S, Thalib L, Williams GM. Advances in the meta-analysis of heterogeneous clinical trials I: The inverse variance heterogeneity model. Contemp Clin Trials. 2015;45(Pt A):130–8. PubMed http://dx.doi.org/10.1016/j.cct.2015.05.009

7. Doi SA, Barendregt JJ, Khan S, Thalib L, Williams GM. Simulation comparison of the quality effects and random effects methods of meta-analysis. Epidemiology. 2015;26:e42–4. PubMed http://dx.doi.org/10.1097/EDE.0000000000000289

8. Onitilo AA, Doi SAR, Barendreg JJ. Meta-analysis II: interpretation and use of outputs. In: Doi SAR, Williams GM, editors. Methods of clinical epidemiology. New York: Springer; 2013.

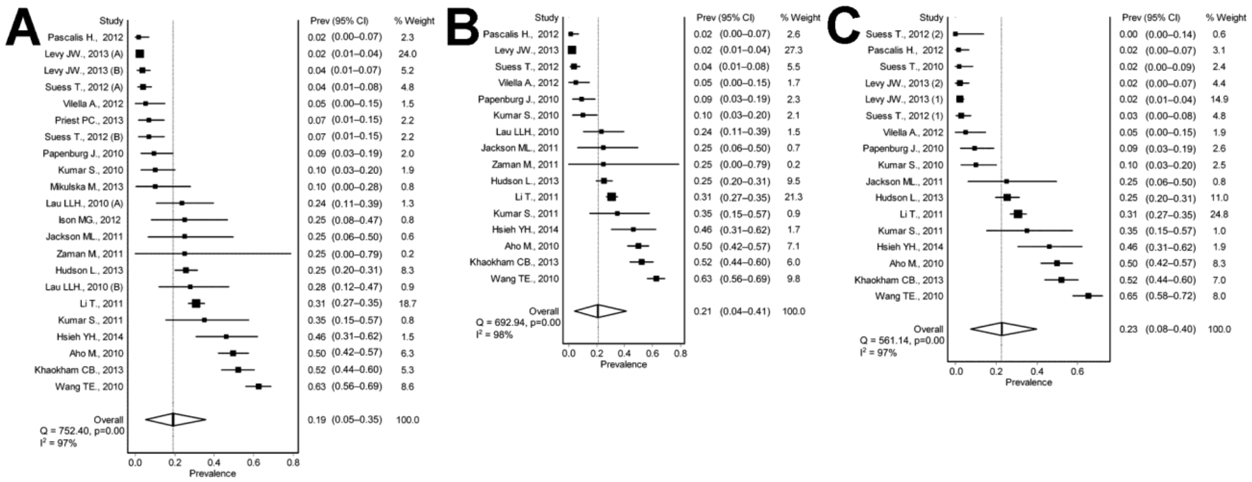

Technical Appendix 2 **Figure 1.** Forest plots for asymptomatic prevalence of A) influenza overall, B) influenza A, and C) influenza A(H1N1) virus infections generated by using the inverse variance heterogeneity model for 55 studies included in systematic review and meta-analysis of asymptomatic and subclinical influenza infection prevalence. Details on these studies are provided in Technical Appendix 1 (http://wwwnc.cdc.gov/EID/article/22/6/15-1080-Techapp1.pdf).

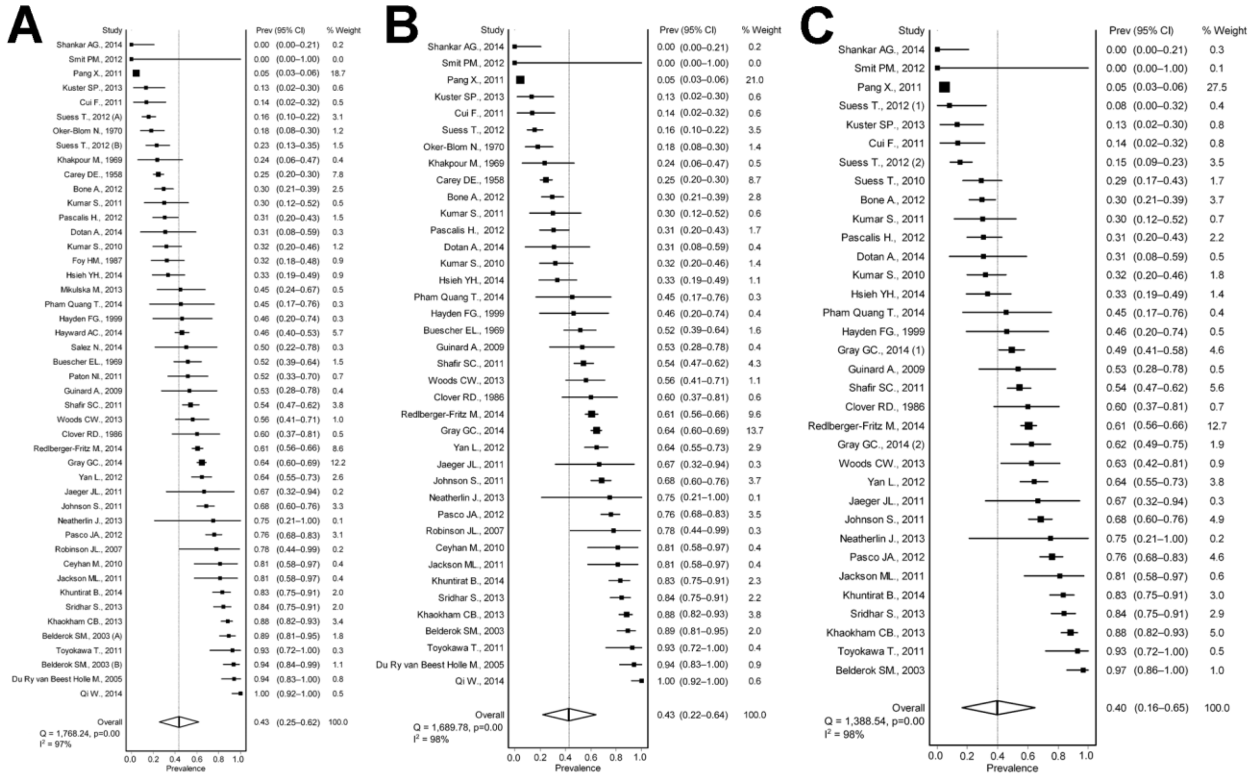

Technical Appendix 2 **Figure 2.** Forest plots for subclinical prevalence of A) influenza overall, B) influenza A, and C) influenza A(H1N1) virus infections generated by using the inverse variance

heterogeneity model for 55 studies included in systematic review and meta-analysis of asymptomatic and subclinical influenza infection prevalence.

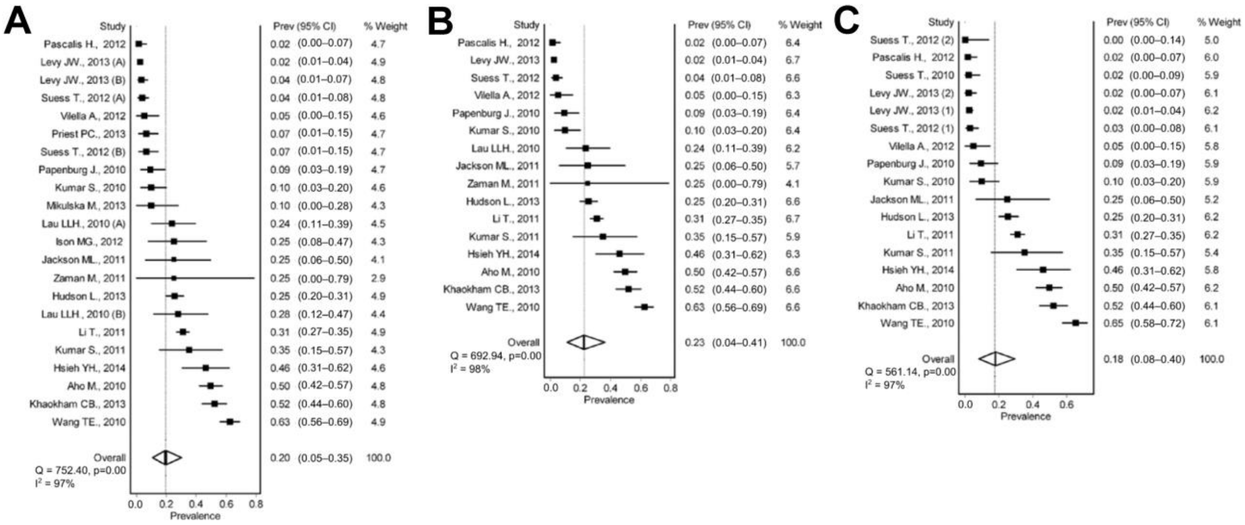

Technical Appendix 2 Figure 3. Forest plots for asymptomatic prevalence of A) influenza overall, B) influenza A, and C) influenza A(H1N1) virus infections generated by using the random effects model for 55 studies included in systematic review and meta-analysis of asymptomatic and subclinical influenza infection prevalence.

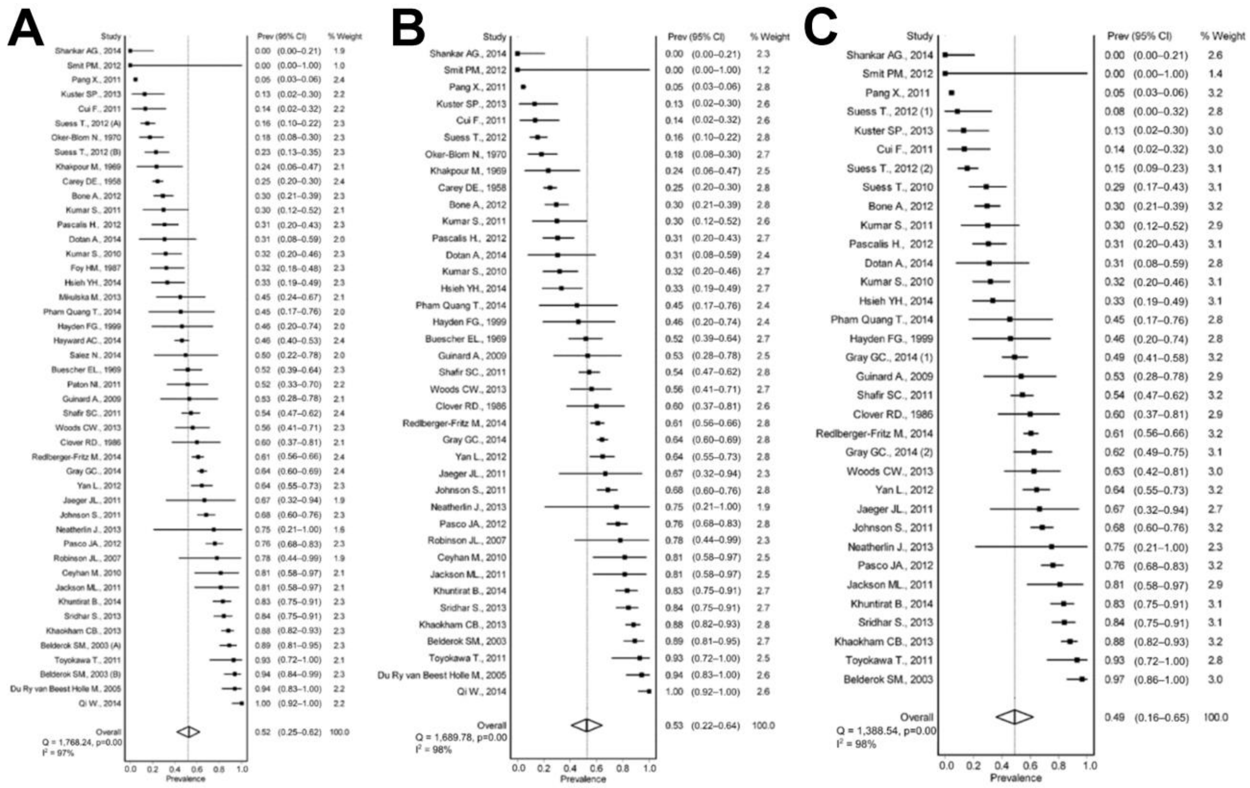

Technical Appendix 2 Figure 4. Forest plots for subclinical prevalence of A) influenza overall, B) influenza A, and C) influenza A(H1N1) virus infections generated by using the random effects model for 55 studies included in systematic review and meta-analysis of asymptomatic and subclinical influenza infection prevalence.
